# A link between problematic social media use and mental health in Greece: Sex and generation differences

**DOI:** 10.3934/publichealth.2025060

**Published:** 2025-12-16

**Authors:** Polyxeni Mangoulia, Aglaia Katsiroumpa, Zoe Katsiroumpa, Evmorfia Koukia, Parisis Gallos, Ioannis Moisoglou, Petros Galanis

**Affiliations:** 1 Laboratory Nursing Counselling and Psychoeducation of Patients and Caregivers, Faculty of Nursing, National and Kapodistrian University of Athens, Athens, Greece; 2 Clinical Epidemiology Laboratory, Faculty of Nursing, National and Kapodistrian University of Athens, Athens, Greece; 3 Faculty of Nursing, University of West Attica, Athens, Greece; 4 Faculty of Nursing, University of Thessaly, Larissa, Greece

**Keywords:** social media, anxiety, depression, sleep quality, sex, generation

## Abstract

Our study aimed to evaluate the association between problematic social media use and mental health. Additionally, we examined sex and generation differences. We performed a cross-sectional study in Greece using a convenience sample. Participants were divided into three generational cohorts: Generation Z (born 1997–2012), Millennials (born 1981–1996), and Generation X (born 1965–1980). To evaluate problematic social media use, we employed the Bergen Social Media Addiction Scale. Anxiety and depression were measured using the Patient Health Questionnaire-4, and the sleep quality was assessed with the Sleep Quality Scale. We developed multivariable linear regression models to control for confounding variables. Our findings revealed a positive correlation between problematic social media use and anxiety, which was unaffected by sex or generation. Additionally, a positive link was found between problematic social media use and depression, with a stronger association observed in Generation Z and Millennials. Moreover, our multivariable models indicated a negative relationship between problematic social media use and sleep quality, which was more pronounced among males and Millennials. In summary, our results underscore the link between problematic social media use and mental health issues. Policymakers, stakeholders, and healthcare professionals should devise and implement suitable interventions to mitigate the adverse effects of problematic social media use.

## 1. Introduction

Over the past decade, the growth spurt of social networking sites has redefined social interactions. Billions of accounts are active, which enables people to use sites such as Facebook, Instagram, and TikTok to communicate, access information, and make friends [1]. The widespread use of social media has raised increasing concerns about its psychological impact. In response, the term problematic social media use (PSMU) was introduced to describe patterns of excessive or compulsive engagement. This behavior is typically marked by preoccupation with social media (salience), attempts to regulate mood through its use, growing tolerance, withdrawal symptoms when not using it, and conflicts that arise from its interference with daily life [2],[3]. Although it cannot be formally considered a mental disorder, PSMU has earned a behavioral addiction framework and is known to have a wider array of mental health outcomes, such as depression, anxiety, and sleep disturbances [4],[5].

Meta-analyses and systematic reviews indicated that social media use and well-being somehow form a complex relationship. Most often, the measurement of use by time or frequency checking shows small (*r* = 0.10–0.17) and often non-significant correlations with the symptoms of depression [1],[6]. In contrast, studies that focused on PSMU showed moderate positive association correlations of PSMU with depression and anxiety [7]. Similar to that, one systematic review on problematic TikTok use found significant relationships with depression (*β* = 0.32), anxiety (*β* = 0.41), and impairments in sleep, body image, and distress intolerance [8]. All of these findings indicate that the amount of time exposed is not the most damaging, but rather the compulsive and addictive use of TikTok.

Individual studies revealed both the dangers and prospects for benefits. Cross-sectional studies found that higher PSMU scores correlate with higher anxiety and depression and that a large emotional investment in social media significantly predicts higher chances of these symptoms than does time spent online [9]. Fear of Missing Out (FOMO) contributes to late-night use and delayed sleep onset. Importantly, Greek studies echo these findings: Katsiroumpa et al. [10] observed that problematic TikTok use was linked to shorter sleep and increased daytime sleepiness in adults; and Bilali et al. [11] reported that among adolescents, problematic use predicted sleepiness and, particularly in boys, anxiety and depressive symptoms. These studies underline the need for country-specific data.

Disruption in sleep could be underlying some association between PSMU and mental health [12]. Comparisons may enhance with an exposure to idealized content, whereas peer conflict or cyberbullying may heighten anxiety. Rumination might further cause some users to intensify depressive symptoms by being unable to detach from the online world. Although there is some evidence that social media use serves certain psychological needs (i.e., to belong and connect) and may, in controlled use, be associated with well-being [13],[14], such benefits seem far less evident where its use is excessive or compulsive.

Currently, social media engagement is widespread among adolescents and young adults. European surveys report that more than three-quarters of 13–16-year-olds hold an online profile, while its usage approaches universality in older adolescents [15],[16]. Although online communication may support social development, it also exposes individuals to FoMO-driven behaviors, social comparison and cyberbullying—factors associated with poor psychological outcomes [17]. The displacement theory further suggests that digital engagement may reduce face-to-face interactions or physical activities, with implications for well-being [18]. Systematic evidence indicates that general social media use tends to show small associations with mental health, while PSMU demonstrates stronger, clinically more relevant patterns [19]. International literature is expanding, although evidence from Greece remains limited. Most research views university students or certain platforms without considering broader age groups. As of now, there has not been any large-scale study that generationally examined PSMU. This gap is noteworthy since Generation Z and Millennials are digital natives socialized in media-rich environments, whereas Generation X later adopted digital technologies [20]. Additionally, sex differences may be relevant, although findings appeared inconsistent across studies [6],[21]. Generational categories are typically defined as 1997–2012 for Generation Z, 1981–1996 for Millennials, and 1965–1980 for Generation X [22],[23], and these groups developmentally differ in terms of their exposure to digital media, which may influence their vulnerability to problematic engagement. The current study explores the associations between PSMU, anxiety, depression, and sleep quality in a large Greek Generation Z, Millennial, and Generation X sample. We hypothesize that PSMU demonstrates a positive association with anxious and depressive symptoms, and a negative association with the quality of sleep, with these relationships possibly differing according to generation and gender. This study aims to achieve a more nuanced understanding of the grave risks and congenial benefits accruing from social media use within the Greek settings by employing validated measures and positioning its findings among the greater literature.

## 2. Materials and methods

### 2.1. Study design

We conducted a web-based cross-sectional study in Greece, thereby utilizing an online questionnaire created via Google Forms and distributed through social media platforms. Specifically, we posted an invitation to join our study on Facebook, Instagram, TikTok, and LinkedIn. Interested participants accepted the invitation and completed the questionnaire. The participants had to be adults over 18 years of age. We informed the participants about the design of the study and their ability to exit the survey by closing their web browser. Additionally, our contact information was provided. We implemented a prior-completion check to preserve the data integrity; respondents that indicated previous participation were removed, thus leaving a convenience sample. Data collection took place from January to March 2025. We adhered to the Reporting of Observational Studies in Epidemiology (STROBE) guidelines for our study [24].

We used G*Power, v.3.1.9.2, to calculate our sample size. Considering a small effect size between PSMU, anxiety, depression, and sleep quality (*f^2^* = 0.02), the five independent variables, a confidence level of 95%, and a margin error of 5%, we needed at least 995 participants. In this formula, we used the fixed model, *R^2^* deviation from the zero.

### 2.2. Measurements

We employed the Bergen Social Media Addiction Scale (BSMAS) to assess PSMU [25]. This scale is comprised of six items that reflect essential addiction components: salience, mood modification, tolerance, withdrawal symptoms, conflict, and relapse. The BSMAS measures behaviors related to PSMU over a year, with each of the six items rated on a 5-point Likert scale from 1 (very rarely) to 5 (very often). The BSMAS is structured as a unifactorial model, allowing total scores to range from 6 to 30, where higher scores signify more severe PSMU [25],[26]. Sample items include statements such as “You spend a lot of time thinking about social media or planning how to use it” and “You feel an urge to use social media more and more”. We utilized the validated Greek version of the BSMAS [27],[28], which demonstrated excellent internal reliability in our study (Cronbach's *α* = 0.867).

We assessed anxiety and depression using the Patient Health Questionnaire-4 (PHQ-4), which consists of four questions: two for anxiety and two for depression [29]. The responses are recorded on a four-point Likert scale ranging from 0 (not at all) to 3 (nearly every day). The scores for both factors can vary from 0 to 6, with higher scores indicating more severe anxiety and depressive symptoms. A score of 3 or above signifies elevated levels of anxiety and depression. We used the Greek version of the PHQ-4 [30]. In our study, the Cronbach's alpha for the PHQ-4 was 0.849, with 0.790 for anxiety and 0.782 for depression.

Sleep quality was assessed using the Sleep Quality Scale (SQS) [31]. The participants rated their overall sleep quality over the past week on a visual analogue scale, thereby selecting an integer from 0 (terrible sleep quality) to 10 (excellent sleep quality). The SQS developers suggested the following cut-off points: 0 = terrible, 1–3 = poor, 4–6 = fair, 7–9 = good, and 10 = excellent sleep quality [31]. When evaluating their sleep quality, the participants considered factors such as the number of hours spent sleeping, the ease of falling asleep, the frequency of waking during the night (excluding bathroom trips), instances of waking earlier than necessary, and how refreshing their sleep felt.

We considered four potential confounding variables: educational level (ranging from elementary school to PhD), socioeconomic status, daily social media usage, and the total number of social media accounts. Socioeconomic status was measured with a straightforward question: “How do you consider your socioeconomic status?”, with responses to be from 0 (lowest level) to 10 (highest level).

### 2.3. Ethical issues

We informed the participants about the study design, and those who agreed were permitted to fill out the questionnaire, thus providing informed consent. Additionally, no identifying information was obtained, thus preserving both voluntariness and anonymity. The study adhered to the Declaration of Helsinki guidelines [32], and the protocol received approval from the Ethics Committee of the Faculty of Nursing, National and Kapodistrian University of Athens (approval number; 05, October 10; 2024).

### 2.4. Statistical analysis

We summarized the categorical variables as counts and percentages, and the continuous variables using mean, standard deviation (SD), median, interquartile range, skewness, and kurtosis. The Kolmogorov-Smirnov test and Q-Q plots were employed to evaluate the distribution of continuous variables. PSMU was treated as the independent variable. Our dependent variables included scores for anxiety, depression, and sleep quality. Variables such as education level, socioeconomic status, daily social media usage, and the total number of social media accounts were considered as potential confounders. Given continuous, normally distributed dependent variables, a linear regression was employed; unadjusted and adjusted b estimates, 95% confidence intervals (*CI*), and *p*-values were reported. All multivariable models were adjusted to account for the aforementioned confounders. To check for multicollinearity in the multivariable models, we used variance inflation factors (VIFs), with values over 4 indicating multicollinearity [33]. The VIFs for the final models ranged from 1.071 to 1.535, thus indicating no multicollinearity issues. Additionally, we performed a stratification analysis to explore differences by sex and generation. The participants were categorized into three generational groups (Dimock, 2019): Generation Z (born 1997–2012), Millennials (born 1981–1996), and Generation X (born 1965–1980). Additionally, we conducted independent samples *t*-tests to examine differences in social media usage and study scales between genders and across the three generations. For the generational analysis, we first conducted an analysis of variance, followed by independent samples *t*-tests between two groups with a Bonferroni correction. Pearson's correlation coefficient was calculated to evaluate relationships between the continuous variables. *P*-values less than 0.05 were considered statistically significant. We used the IBM SPSS, v. 28.0, for the analysis.

## 3. Results

### 3.1. Demographic characteristics

Our sample consisted of 1033 individuals. In our sample, 75.4% were females and 24.6% were males. The average age was 31.1 years. Generation Z was 53.6% of our sample, followed by Millennials at 28.6%, and Generation X at 17.8%. Table 1 shows the demographic data of the sample.

**Table 1.** Study population.

**Characteristics**	***N***	**%**
Sex		
Females	779	75.4
Males	254	24.6
Age^a^	31.1	12.4
Age categories		
Generation Z	554	53.6
Millennials	295	28.6
Generation X	184	17.8
Educational level		
High school	409	39.6
Bachelor degree	373	36.1
MSc diploma	229	22.2
PhD diploma	22	2.1
Socioeconomic status^a^	6.2	1.5

Note: ^a^ mean, standard deviation.

### 3.2. Social media characteristics

On average, the participants dedicated 3.3 hours each day to social media. Females spent a daily average of 3.4 hours, while males averaged 3.3 hours (*p*-value = 0.356). Generation Z averaged 4.0 hours, Millennials averaged 2.9 hours, and Generation X averaged 2.1 hours on social media each day (*p*-value < 0.001 for differences among all groups).

The average number of accounts was 3.6. Females averaged 3.6 accounts, while males had 3.9 (*p*-value = 0.011). Generation Z averaged 3.8 accounts, Millennials averaged 3.7 accounts, and Generation X averaged 3.1 accounts (*p*-value < 0.001 for differences between Generation Z and X, and between Millennials and Generation X).

Table 2 presents the daily social media use and social media accounts of our participants stratified by sex and generation.

**Table 2.** Social media use and social media accounts of our participants.

	**Sex**		**Generation**			**Total**
	**Females (*n* = 779)**	**Males (*n* = 254)**	**Generation Z (*n* = 554)**	**Millennials (*n* = 295)**	**Generation X (*n* = 184)**	
Social media use per day (hours)						
Mean	3.4	3.3	4.0	2.9	2.1	3.3
Standard deviation	1.9	1.8	1.8	1.7	1.4	1.9
*p*-value^a^	0.356		<0.001^b^ for all comparisons between the three groups		
Social media accounts						
Mean	3.6	3.9	3.8	3.7	3.1	3.6
Standard deviation	1.5	1.7	1.5	1.5	1.4	1.5
*p*-value^a^	0.011		<0.001^b^ for comparisons between Generation Z and X, and between Millennials and Generation X		

Note: ^a^ independent samples *t*-test; ^b^ *p*-values after Bonferroni correction.

### 3.3. Study scales

The mean BSMAS score was 12.12 (*SD*; 4.92). No significant difference was found in BSMAS scores between females (mean; 12.08, *SD*; 4.81) and males (mean; 12.23, *SD*; 5.22), (*p*-value = 0.690). Generation Z (mean; 13.93, *SD*; 5.01) had higher BSMAS scores than Millennials (mean; 10.84, *SD*; 4.23) and Generation X (mean; 8.70, *SD*; 2.71), (*p*-value < 0.001 in all cases).

The mean score on PHQ-4 was 4.25 (*SD*; 2.95), with a mean anxiety score of 2.38 (*SD*; 1.62) and a mean depression score of 1.87 (*SD*; 1.61). One-third of the participants (35.0%, *n* = 362) had an anxiety score of 3 or higher, thus indicating significant anxiety issues. Additionally, one-fourth (24.9%, *n* = 257) had a depression score ≥3, thus indicating high levels of depressive symptoms.

The mean score on the SQS was 5.73 (*SD*; 2.26). Sleep quality was rated as terrible by 1.5% (*n* = 16) of participants, poor by 17.1% (*n* = 177), fair by 39.1% (*n* = 403), good by 33.6% (*n* = 409), and excellent by 2.7% (*n* = 28).

Descriptive statistics for the study scales are shown in Table 3.

**Table 3.** Descriptive statistics for our study scales (*N* = 1033).

**Scale**	**Mean**	**Standard deviation**	**Median**	**Interquartile range**	**Skewness**	**Kurtosis**
Bergen social media addiction scale	12.12	4.92	11.00	7.00	0.79	−0.10
Patient health Questionnaire-4	4.25	2.95	4.00	4.00	0.84	0.20
Anxiety	2.38	1.62	2.00	2.00	0.72	0.11
Depression	1.87	1.61	2.00	1.00	0.90	0.30
Sleep quality scale	5.73	2.26	6.00	3.00	−0.32	−0.52

### 3.4. Correlation between study scales

Our findings indicate a positive correlation between the social media addiction scores and the anxiety scores, with a stronger correlation observed among males and Generation Z. Similarly, a positive correlation was found between the social media addiction scores and the depression scores, again stronger among males and Generation Z. A negative correlation was observed between the social media addiction scores and the sleep quality scores, with a stronger correlation among males, Generation Z, and Millennials.

Table 4 shows the correlations between the social media addiction scores and the anxiety scores, depression scores, and sleep quality scores.

**Table 4.** Pearson's correlation coefficients between social media addiction score, and anxiety score, depression score and sleep quality score (*N* = 1033).

	**Anxiety score**	**Depression score**	**Sleep quality score**
Full sample (*n* = 1033)	0.282*	0.381*	−0.197*
Females (*n* = 779)	0.250*	0.355*	−0.157*
Males (*n* = 254)	0.392*	0.454*	−0.299*
Generation Z (*n* = 554)	0.311*	0.427*	−0.206*
Millennials (*n* = 295)	0.240*	0.340*	−0.246**
Generation X (*n* = 184)	0.273*	0.270*	−0.145

Note: Coefficients are adjusted for educational level, socioeconomic status, social media use per day, and social media accounts. * *p*-value < 0.001.

### 3.5. Association between problematic social media use and anxiety

We identified a positive correlation between PSMU and anxiety across the entire sample (adjusted coefficient beta = 0.104, 95% *CI* = 0.083 to 0.126, *p*-value < 0.001). A stratified analysis revealed that this association was similar among females (adjusted coefficient beta = 0.097, 95% *CI* = 0.070 to 0.124, *p*-value < 0.001) and males (adjusted coefficient beta = 0.127, 95% *CI* = 0.090 to 1.165, *p*-value < 0.001), and no differences were observed between the generations. Specifically, the association between PSMU and anxiety was consistent across Generation Z (adjusted coefficient beta = 0.110, 95% *CI* = 0.083 to 0.138, *p*-value < 0.001), Millennials (adjusted coefficient beta = 0.099, 95% *CI* = 0.052 to 0.146, *p*-value < 0.001), and Generation X (adjusted coefficient beta = 0.123, 95% *CI* = 0.061 to 0.184, *p*-value < 0.001). See Table 5.

**Table 5.** Linear regression models with anxiety score as the dependent variable.

**Predictor: BSMAS**	**Univariate model**			**Multivariable model^a^**					
	**Unadjusted coefficient beta**	**95% *CI* for beta**	***p*-value**	**Adjusted coefficient beta**	**95% *CI* for beta**	***p*-value**	**VIF**	***R^2^* (%)**	***p*-value for ANOVA**
Full sample (*n* = 1033)	0.164	0.526 to 1.006	<0.001	0.104	0.083 to 0.126	<0.001	1.478	20.4	<0.001
Females (*n* = 779)	0.126	0.104 to 0.147	<0.001	0.097	0.070 to 0.124	<0.001	1.535	16.3	<0.001
Males (*n* = 254)	0.155	0.121 to 1.188	<0.001	0.127	0.090 to 1.165	<0.001	1.372	31.4	<0.001
Generation Z (*n* = 554)	0.117	0.091 to 0.142	<0.001	0.110	0.083 to 0.138	<0.001	1.204	16.1	<0.001
Millennials (*n* = 295)	0.130	0.089 to 0.171	<0.001	0.099	0.052 to 0.146	<0.001	1.364	14.1	<0.001
Generation X (*n* = 184)	0.096	0.037 to 0.156	0.002	0.123	0.061 to 0.184	<0.001	1.184	13.9	<0.001

Note: ^a^ Multivariable models are adjusted for educational level, socioeconomic status, social media use per day, and social media accounts. *CI*: confidence interval, BSMAS: Bergen Social Media Addiction Scale, VIF: variance inflation factor.

### 3.6. Association between problematic social media use and depression

The final multivariable linear regression model in the full sample showed a positive association between PSMU and depression (adjusted coefficient beta = 0.145, 95% *CI* = 0.123 to 0.166, *p*-value < 0.001). After stratification, this positive association was consistent among both females (adjusted coefficient beta = 0.140, 95% *CI* = 0.114 to 0.165, *p*-value < 0.001) and males (adjusted coefficient beta = 0.158, 95% *CI* = 0.119 to 0.197, *p*-value < 0.001). Additionally, our stratified analysis revealed that the link between PSMU and depression was stronger in Generation Z (adjusted coefficient beta = 0.152, 95% *CI* = 0.124 to 0.180, *p*-value < 0.001) and Millennials (adjusted coefficient beta = 0.140, 95% *CI* = 0.094 to 0.185, *p*-value < 0.001) compared to Generation X (adjusted coefficient beta = 0.111, 95% *CI* = 0.053 to 0.170, *p*-value < 0.001). Table 6 shows the linear regression models with the depression score as the dependent variable.

**Table 6.** Linear regression models with depression score as the dependent variable.

**Predictor: BSMAS**	**Univariate model**			**Multivariable model^a^**					
	**Unadjusted coefficient beta**	**95% *CI* for beta**	***p*-value**	**Adjusted coefficient beta**	**95% *CI* for beta**	***p*-value**	**VIF**	***R^2^* (%)**	***p*-value for ANOVA**
Full sample (*n* = 1033)	0.149	0.132 to 0.167	<0.001	0.145	0.123 to 0.166	<0.001	1.478	21.7	<0.001
Females (*n* = 779)	0.148	0.127 to 0.169	<0.001	0.140	0.114 to 0.165	<0.001	1.535	19.7	<0.001
Males (*n* = 254)	0.155	0.121 to 0.189	<0.001	0.158	0.119 to 0.197	<0.001	1.372	27.6	<0.001
Generation Z (*n* = 554)	0.148	0.122 to 0.174	<0.001	0.152	0.124 to 0.180	<0.001	1.204	20.0	<0.001
Millennials (*n* = 295)	0.153	0.114 to 0.192	<0.001	0.140	0.094 to 0.185	<0.001	1.364	17.6	<0.001
Generation X (*n* = 184)	0.091	0.036 to 0.147	0.001	0.111	0.053 to 0.170	<0.001	1.184	10.0	<0.001

Note: ^a^ Multivariable models are adjusted for educational level, socioeconomic status, social media use per day, and social media accounts. *CI*: confidence interval, BSMAS: Bergen Social Media Addiction Scale, VIF: variance inflation factor.

### 3.7. Association between problematic social media use and sleep quality

After adjusting confounders, we discovered a negative association between PSMU and sleep quality score (adjusted coefficient beta = −0.107, 95% *CI* = −0.139 to −0.074, *p*-value < 0.001). This association was more pronounced among males (adjusted coefficient beta = −0.149, 95% *CI* = −0.209 to −0.089, *p*-value < 0.001) and Millennials (adjusted coefficient beta = −0.157, 95% *CI* = −0.228 to −0.086, *p*-value < 0.001). Table 7 shows the linear regression models with the sleep quality score as the dependent variable.

**Table 7.** Linear regression models with sleep quality score as the dependent variable.

**Predictor: BSMAS**	**Univariate model**			**Multivariable model^a^**					
	**Unadjusted coefficient beta**	**95% *CI* for beta**	***p*-value**	**Adjusted coefficient beta**	**95% *CI* for beta**	***p*-value**	**VIF**	***R^2^* (%)**	***p*-value for ANOVA**
Full sample (*n* = 1033)	−0.114	−0.141 to −0.087	<0.001	−0.107	−0.139 to −0.074	<0.001	1.478	9.1	<0.001
Females (*n* = 779)	−0.089	−0.121 to −0.057	<0.001	−0.087	−0.126 to −0.048	<0.001	1.535	7.6	<0.001
Males (*n* = 254)	−0.178	−0.230 to −0.126	<0.001	−0.149	−0.209 to −0.089	<0.001	1.372	18.7	<0.001
Generation Z (*n* = 554)	−0.086	−0.123 to −0.049	<0.001	−0.098	−0.137 to −0.058	<0.001	1.204	6.1	<0.001
Millennials (*n* = 295)	−0.147	−0.210 to −0.084	<0.001	−0.157	−0.228 to −0.086	<0.001	1.364	12.6	<0.001
Generation X (*n* = 184)	−0.112	−0.214 to −0.010	0.032	−0.119	−0.230 to −0.008	0.035	1.071	2.6	0.098

Note: ^a^ Multivariable models are adjusted for educational level, socioeconomic status, social media use per day, and social media accounts. *CI*: confidence interval, BSMAS: Bergen Social Media Addiction Scale, VIF: variance inflation factor.

## 4. Discussion

### 4.1. Main findings and comparison with prior evidence

The present study examined PSMU among cohorts spanning three generations in Greece, with the associations between PSMU and anxiety, depression, and sleep quality. There was a moderate positive correlation between PSMU and the anxiety and depressive symptoms among all participants. This pattern is consistent with recent meta-analytic work which demonstrated that compulsive or addiction-like engagement manifests a stronger relationship to mental health outcomes than do simple measures of time spent online. In fact, moderate positive correlations between PSMU and depression and anxiety were reported in the studies of Ahmed et al. [7] and Galanis et al. [8].

In a recent meta-analysis of 209 studies, problematic social network use exhibited moderate positive correlations with generalized anxiety (*r* = 0.39), social anxiety (*r* = 0.44), attachment anxiety (*r* = 0.35) and FOMO (*r* = 0.50), with effect sizes varying by region, gender, and measurement instrument [34]. These effect sizes were markedly greater than those usually associated with general social media use, where correlations with depressive symptoms typically appeared small (*r* = 0.10–0.17) and inconsistent when use was assessed in terms of time or checking frequency [1],[6], thus suggesting that compulsive involvement rather than mere exposure is more harmful. Systematic reviews reported that although only a small portion of users meet PSMU criteria, those who do demonstrate significantly greater likelihoods of depressive, anxious, and stress-related symptoms [4],[19], thus aligning with cross-sectional findings that link high PSMU and emotional investments with increased risks for anxiety and depression [9]. These associations may operate through mechanisms such as rumination and low self-esteem [35], and can differ across cultures and age groups. Greek research further supports this relationship, with Bilali et al. [11] documenting that problematic TikTok use among adolescents predicted anxiety and depressive symptoms, particularly in boys, while our data found consistent anxiety-related effects across sexes but stronger depression effects in Gen Z and Millennials. In terms of sleep-related outcomes, both our findings and those of Katsiroumpa et al. [10] pointed out the negative relationship of problem use with sleep quality. They found in their Greek young adult study that higher TikTok addiction scores were related to decreased levels of nighttime sleep and increased levels of daytime sleepiness, just as we found poorer sleep quality associated with higher levels of PSMU, especially among males and Millennials. These converging patterns would amplify the more general linkage between engaged behavior in social media and the impairment in sleep hygiene across ages.

### 4.2. Generational differences and socio demographic patterns

The results revealed generational variation, with PSMU showing stronger associations with depressive symptoms in Millennials and Generation Z compared with Generation X, whereas anxiety effects were more similar across groups. Younger generations tend to use social media more intensively and integrate online interactions in core social functioning, making them more susceptible to upward social comparison, FOMO-driven checking, and reward-based reinforcement patterns [15],[16],[20]. This aligns with previous work that showed an increased risk for problematic use in younger populations, as well as Croatian longitudinal evidence that linked high engagement to life satisfaction shifts [17]. In contrast, older adults may rely more heavily on offline coping resources and social support, thereby buffering emotional impact.

Sex differences were minimal overall, which is consistent with meta-analytic evidence that gender does not reliably moderate the association between PSMU and depression or anxiety [6]. However, usage motives and emotional response patterns may differ in nuance, with men demonstrating more entertainment-focused use and women reporting more interpersonal sensitivity and negative emotionality [21]. Additionally, U.S. adolescent research indicated greater body-related pressures and cyberbullying exposure among girls, while boys experienced more peer-bonding use [36]. This suggests that qualitative aspects of use—not sex itself—may be more relevant in shaping mental health outcomes.

### 4.3. Sleep quality and circadian disruptions

There are several reasons why PSMU can co-occur with anxiety, sadness, and sleep problems. Cognitive behavioral theories suggest that maladaptive cognitions (such rumination and negative self-evaluation) and personality traits such as low self-esteem moderate the link between social media addiction and depression [35]. While upward social comparison increases the negative affect, FOMO leads to continual monitoring and can worsen anxiety [12],[36]. From the perspective of platform affordances, the exposure to posts on sports, friends, or family has a positive association with sleep, while viewing women or strangers has a negative association with sleep quality [37].

Longitudinal evidence holds that the association between PSMU and sleep could be affected by depression and stress [37], which further calls for future studies to determine if bettering sleep hygiene decreases the vulnerability versus whether emotional distress is the main driver for both PSMU and sleep concerns. Katsiroumpa et al. [10] would further support this notion, as they found that increased TikTok addiction was related to decreased amount of sleep and an increased amount of daytime sleepiness among young adults. In support of our findings, TikTok use appears to be connected with the poorer quality of sleep among Millennials, thus indicating that this is a form of digital addiction that adds to poor restorative sleep.

### 4.4. Mechanisms and theoretical perspectives

Several theories can explain how PSMU occurs alongside anxiety, depression, and disturbances in sleep. Cognitive behavioral models argue that social media addiction becomes linked to depression through the mediation of maladaptive cognition (e.g., rumination, negative self-evaluation) and personality traits such as low self-esteem [35]. Thus, FOMO drives endless checking, which may worsen anxiety, while upward social comparisons enhance the negative effects [12],[36]. The displaced activities theory argues that time spent in sedentary online activities displaces time for social interactions, physical activity, or sleep, thus indirectly harming mental health [18]. Meanwhile, the uses and gratifications framework remind us that people actively select media to fulfill certain psychological needs; thus, those with pre-existing mental health problems may be using social media even more intensively as a means of seeking out support, distraction, or validation [38]. The actual directionality of effects is still uncertain; our cross-sectional design will not allow disentangling whether PSMU is a cause of or a consequence related to psychological distress. Longitudinal or experimental studies should assess the above-mentioned mechanisms, including an objective measurement of use and sleep, and assess whether interventions targeting cognitions (e.g., reducing rumination) or behaviors (e.g., limiting nighttime use) would lead to better outcomes.

### 4.5. Limitations

Our research encountered several limitations. First, although we employed valid instruments to evaluate PSMU, anxiety, depression, and sleep quality, the participant's answers might have been swayed by social desirability bias, thus potentially leading to information bias in our findings. Furthermore, information bias could stem from measuring confounding factors, such as relying on self-reported data for socioeconomic status. Second, we used a convenience sample, and thus, our sample mainly consisted of females, which could introduce selection bias due to this gender disparity. Future work should apply random (probability) sampling to improve representativeness. Third, as our study was cross-sectional, we could not establish a causal relationship between PSMU and anxiety, depression, and sleep quality. Therefore, it remains uncertain whether PSMU affects anxiety, depression, and sleep quality, or if these issues pre-exist and lead to increased social media use. Longitudinal studies that explore the connection between PSMU and these variables could offer valuable insights. Fourth, we considered several confounders in our study. However, other factors might still confound the relationship between PSMU, anxiety, depression, and sleep quality. Future research should aim to eliminate additional confounders, such as personality traits, family relationships, and sleep patterns. Fifth, investigating potential mediators in the relationship between PSMU, anxiety, depression, and sleep quality could further enhance our understanding of social media's impact. Sixth, we should recognize that our sample size was not powered for sex and age generations. Thus, our findings regarding sex and age differences should be interpreted with caution. Future studies with bigger and more representative samples could add significant information. Finally, we recruited our participants from social media, and thus, they were social media users. This approach excluded individuals who do not use social media or those who use social media less frequently. Therefore, a selection bias was introduced in our study that can result in a potential overestimation of PSMU. Scholars in future studies could employ a different approach to achieve their sample by including individuals who do not or less frequently use social media to further validate our findings.

## 5. Conclusions

This work confirms the strong association between PSMU and adverse mental health outcomes (i.e., higher levels of anxiety, depression and decreased sleep quality). Although the associations between PSMU and anxiety were similar across generations and sex, its relationship to depression was stronger in Generation Z and Millennials; furthermore, it had a pronounced negative effect on sleep quality in males and Millennials. These results suggest that the psychological implications of social media use are influenced by individual practices as well as demographic characteristics. Targeted, age- and gender-sensitive interventions are needed to address compulsive digital engagement and to support mental well-being in the context of pervasive online connectivity.

## Data availability statement

Data are openly available in FigShare at https://doi.org/10.6084/m9.figshare.28903820.

## Use of AI tools declaration

The authors declare they have not used Artificial Intelligence (AI) tools in the creation of this article.

## References

[1] Valkenburg PM, Meier A, Beyens I (2022). Social media use and its impact on adolescent mental health: An umbrella review of the evidence. Curr Opin Psychol.

[2] Andreassen CS, Pallesen S (2014). Social network site addiction—an overview. Curr Pharm Des.

[3] Griffiths M (2005). A “components” model of addiction within a biopsychosocial framework. J Subst Use.

[4] Cataldo I, Lepri B, Neoh MJY (2021). Social media usage and development of psychiatric disorders in childhood and adolescence: A systematic review. Front Psychiatry.

[5] Weinstein AM (2023). Problematic social networking site use: Effects on mental health and the brain. Front Psychiatry.

[6] Vahedi Z, Zannella L (2021). The association between self reported depressive symptoms and the use of social networking sites: A meta-analysis. Curr Psychol.

[7] Ahmed O, Walsh EI, Dawel A (2024). Social media use, mental health and sleep: A systematic review with meta analyses. J Affect Disord.

[8] Galanis P, Katsiroumpa A, Katsiroumpa Z (2025). Association between problematic TikTok use and mental health: A systematic review and meta analysis. AIMS Public Health.

[9] Alsunni AA, Latif R (2020). Higher emotional investment in social media is related to anxiety and depression. J Taibah Univ Med Sci.

[10] Katsiroumpa A, Moisoglou I, Gallos P (2025). Problematik TikTok use and its association with poor sleep among greek young adults. Psychiatry Int.

[11] Bilali A, Katsiroumpa A, Koutelekos I (2025). Association between TikTok use and anxiety, depression, and sleepiness among adolescents: A cross-sectional study in Greece. Pediatr Rep.

[12] Scott H, Cleland Woods H (2018). Fear of missing out and sleep: Cognitive behavioural factors in adolescents' nighttime social media use. J Adolesc.

[13] Houghton D, Pressey A, Istanbulluoglu D (2020). Who needs social networking? An empirical enquiry into the capability of Facebook to meet human needs and increase satisfaction with life. Comput Hum Behav.

[14] Hartanto A, Yong JC, Toh WX (2020). Cognitive, social, emotional, and subjective health benefits of computer use in adults: A 9-year longitudinal study from the Midlife in the United States (MIDUS). Comput Hum Behav.

[15] Livingstone S, Ólafsson K, Staksrud E (2011). Social networking, age and privacy.

[16] Tsitsika AK, Tzavela EC, Janikian M (2014). Online social networking in adolescence: Patterns of use in six European countries and links with psychosocial functioning. J Adolesc Health.

[17] Keresteš G, Štulhofer A (2020). Adolescents' online social network use and life satisfaction: A latent growth curve modelling approach. Comput Hum Behav.

[18] Karim F, Oyewande AA, Abdalla LF (2020). Social media use and its connection to mental health: A systematic review. Cureus.

[19] Keles B, McCrae N, Grealish A (2020). A systematic review: the influence of social media on depression, anxiety and psychological distress in adolescents. Int J Adolesc Youth.

[20] Crone EA, Konijn EA (2018). Media use and brain development during adolescence. Nat Commun.

[21] Sobieraj S, Kraemer NC (2020). Similarities and differences between genders in the usage of computer with different levels of technological complexity. Comput Hum Behav.

[22] Cantrell MS (2020). Generations at a glance. IFAS, University of Florida.

[23] Dimock M (2019). Defining generations: Where Millennials end and Generation Z begins. Pew Research Center.

[24] Vandenbroucke JP, von Elm E, Altman DG (2007). Strengthening the reporting of observational studies in epidemiology (STROBE): Explanation and elaboration for the STROBE initiative. PLoS Med.

[25] Andreassen CS, Billieux J, Griffiths MD (2016). The relationship between addictive use of social media and video games and symptoms of psychiatric disorders: A large-scale cross-sectional study. Psychol Addict Behav.

[26] Monacis L, de Palo V, Griffiths MD (2017). Social networking addiction, attachment style, and validation of the Italian version of the Bergen Social Media Addiction Scale. J Behav Addict.

[27] Dadiotis A, Bacopoulou F, Kokka I (2021). Validation of the Greek version of the Bergen Social Media Addiction Scale in undergraduate students. EMBnet J.

[28] Katsiroumpa A, Katsiroumpa Z, Koukia E (2025). Bergen Social Media Addiction Scale: Translation and validation in Greek. Int J Caring Sci.

[29] Kroenke K, Spitzer RL, Williams JBW (2009). An ultra-brief screening scale for anxiety and depression: the PHQ-4. Psychosomatics.

[30] Karekla M, Pilipenko N, Feldman J (2012). Patient Health Questionnaire: Greek language validation and subscale factor structure. Compr Psychiatry.

[31] Snyder E, Cai B, de Muro C (2018). New single-item Sleep Quality Scale: Results of psychometric evaluation in patients with chronic primary insomnia and depression. J Clin Sleep Med.

[32] World Medical Association (2013). World Medical Association Declaration of Helsinki ethical principles for medical research involving human subjects. JAMA.

[33] Kim JH (2019). Multicollinearity and misleading statistical results. Korean J Anesthesiol.

[34] Du M, Zhao C, Hu H (2024). Association between problematic social networking use and anxiety symptoms: A systematic review and meta analysis. BMC Psychol.

[35] Wang P, Wang X, Wu Y (2018). Social networking sites addiction and adolescent depression: A moderated mediation model of rumination and self esteem. Pers Individ Dif.

[36] Kreski N, Platt J, Rutherforda C (2021). Social media use and depression symptoms among United States adolescents. J Adolesc Health.

[37] Bergfeld NS, Van den Bulck J (2021). It's not all about the likes: Social media affordances with nighttime, problematic, and adverse use as predictors of adolescent sleep indicators. Sleep Health.

[38] Deci EL, Ryan RM (2000). The “what” and “why” of goal pursuits: Human needs and the self-determination of behavior. Psychol Inq.

